# Optimized irrigation and fertilization for spring maize under warming and wetting climate in a semi-arid region of China

**DOI:** 10.3389/fpls.2025.1600561

**Published:** 2025-07-02

**Authors:** Hongjuan Zhang, Rui Zhang, Lina Sun, Haolin Li, Yanling Xue, Xia Zhao, Jiahui Liu, Chao Yuan

**Affiliations:** ^1^ College of Water Conservancy and Hydropower Engineering, Gansu Agricultural University, Lanzhou, China; ^2^ Liangzhou District Agricultural Technology Extension Center, Wuwei, China; ^3^ College of Environmental and Energy Engineering, Beijing University of Technology, Beijing, China; ^4^ Zhangye City Water Saving Irrigation Experimental Research Center, Zhangye, China

**Keywords:** RZWQM2 model, spring maize, irrigation quota, fertilizer application rate, climate scenario

## Abstract

Inefficient irrigation and fertilizer practices in spring maize production in a Chinese semi-arid region have led to suboptimal fertilizer utilization and yield limitations. Few studies in this region have adequately incorporated long-term meteorological data to optimize irrigation and fertilizer strategies. In this study, we employed the Root Zone Water Quality Model 2 (RZWQM2) to evaluate and optimize irrigation and fertilizer management practices. The model was calibrated and validated using field experimental data during 2022–2023, including two irrigation levels [75%–95% (I1) and 55%–75% field capacity (I2)] and three fertilizer treatments [234.27 (F1), 157.5 (F2), and 157.5 kg hm^−2^ nitrogen fertilizer (F3), and F3 plus 63 kg hm^−2^ organic fertilizer). The validated model demonstrated excellent performance in simulating key parameters, including soil water content (SWC) [mean relative error (*MRE*) and normalized root mean squared error (*NRMSE*) < 15%, consistency index (*d*) > 0.80], biomass (*d* > 0.85), grain yield (*MRE* < 15%), and NH_4_^+^-N and NO_3_^−^-N contents (*RMSE* < 10 mg kg^−1^, *MRE* and *NRMSE* < 15%, *d* > 0.60), of spring maize in 2022 and 2023. Under simulated climate scenarios, optimal yields of 21.54, 20.78, and 17.57 t hm^−2^ were achieved using a combined application of 60% nitrogen and 40% organic fertilizer across three irrigation quotas. The irrigation quota of 250 m^3^ hm^−2^ demonstrated superior water use efficiency (*WUE*), irrigation water use efficiency (*IWUE*), and partial factor productivity (*PFP*) compared to quotas of 300 and 200 m^3^ hm^−2^. These findings provide valuable insights for developing sustainable irrigation and fertilizer strategies for spring maize production in a semi-arid region of China.

## 1 Introduction

Maize is one of the most important grain crops in the world, accounting for 36.8% of China’s total grain crop planting area and ranking first in production volume (Li et al., 2017; Zhang et al., 2024). Adequate light and heat resources are important factors for spring maize growth in a semi-arid region of China (Kang et al., 2014). However, water resources are scarce in this region, accounting for only approximately 10% of the country’s total water resources, which severely constrains local maize production (Wang et al., 2018). Irrigation and fertilizer are crucial measures to ensure high maize yields in a semi-arid region (Qiang et al., 2019). In the widespread cultivation of maize in a semi-arid region, farmers often rely on over-irrigation and excessive fertilization to achieve high crop yields (Liu and Zhang, 2007), which significantly reduces irrigation and fertilizer use efficiency (Yang et al., 2022). Therefore, optimal irrigation and fertilizer management that achieves both high yields and resource use efficiency is critical to address these challenges and ensure sustainable spring maize production in a semi-arid region of China.

The integrated optimization of irrigation and fertilizer management strategies is considered an effective way to improve irrigation and fertilizer use efficiency and cope with water scarcity in agriculture (Yan et al., 2021). Recent years have witnessed growing scientific efforts to optimize integrated irrigation and fertilizer management protocols in maize cultivation systems, particularly focusing on precision irrigation scheduling and nutrient delivery mechanisms (Zhou et al., 2019), and the combined application of inorganic and organic fertilizers in irrigation and fertilizer research has been increasingly proposed (Baharuddin and Tejowulan, 2021; Ilahi et al., 2020). Meanwhile, soil water content control technology can effectively reduce deep soil water content leakage and nitrogen fertilizer leaching while achieving high yield and water use efficiency (Fang et al., 2008; Panda et al., 2004). Studies have shown that replacing some chemical fertilizers with organic fertilizers can significantly increase maize yield, water use efficiency, and economic benefits while reducing nitrogen fertilizer application (Zhai et al., 2022; Zhou et al., 2022; Wang et al., 2017). Wang et al. (2017) determined that organic fertilizer application improved water use in 50–150-cm soil depth and increased grain yield by 5%–10%. He et al. (2022) found that the application of organic fertilizers increased the yield of wheat and maize by 26.4%–44.6% and 12.5%–40.8%, respectively. However, these studies were conducted over only 2–3 years of field experiments and were unable to determine the optimal irrigation and fertilizer application rates under different climate scenarios.

In recent years, research on the effects of increased temperature and precipitation on crops under global warming scenarios has received increasing attention (Zhang et al., 2021; Ureta et al., 2020). Over the past few decades, climate change, primarily characterized by increases in temperature and precipitation, has severely impacted crop growth and water use in the Northwest (Chen et al., 2015). Liu et al. (2018) studied yield changes in spring wheat and summer maize by organic and inorganic fertilizers through long-term fertilization and climate change. Crop modeling systems have emerged as pivotal methodological frameworks for evaluating climate change effects on agricultural systems, particularly in analyzing yield formation dynamics, irrigation scheduling optimization, and precision nutrient management (Matthews et al., 2013; Bassu et al., 2014). The Root Zone Water Quality Model 2 (RZWQM2) model has been widely evaluated and applied in studying climate impacts on crops. Chen et al. (2019) used the RZWQM2 model to investigate the effects of climate change on cotton yield and crop water demand in extreme drought regions. Liu et al. (2019) qualitatively analyzed the response of grain yields to major climatic variables through the RZWQM2 model and predicted grain yields of maize in the Siping region, Jilin Province, Northeast China, during the period from 1951 to 2015. Ding et al. (2024) developed an optimized nitrogen fertilizer strategy for winter wheat based on precipitation changes using the RZWQM2 model in the Loess Plateau region. Meanwhile, the RZWQM2 model has been widely used by a large number of studies on crop growth, water and salt transport, optimal management of irrigation and fertilizer under climate change, and greenhouse gas emissions from farmland (Zhang et al., 2025; Singh et al., 2025). While substantial modeling efforts have elucidated irrigation–inorganic fertilizer interactions under climate change scenarios, research addressing model-specific irrigation systems coupled with integrated organic–inorganic fertilization remains critically underexplored.

However, most simulations have been conducted using either irrigation or nitrogen fertilizer alone, while studies on the combined effects of irrigation and coupled organic and inorganic fertilizers under climate change scenarios are limited. Consequently, further study is required to assess the performance of the RZWQM2 model and develop optimal management strategies for irrigation and fertilizer application rates for spring maize. The objectives of this study were 1) to evaluate the applicability of the RZWQM2 model for simulating soil water content, biomass, grain yield, and NH_4_^+^-N and NO_3_^−^-N contents of spring maize in a semi-arid region; and 2) to determine an optimal irrigation quota and fertilizer application rate for spring maize in the study area under climate change scenarios.

## 2 Materials and methods

### 2.1 Study site

**Figure 1:**
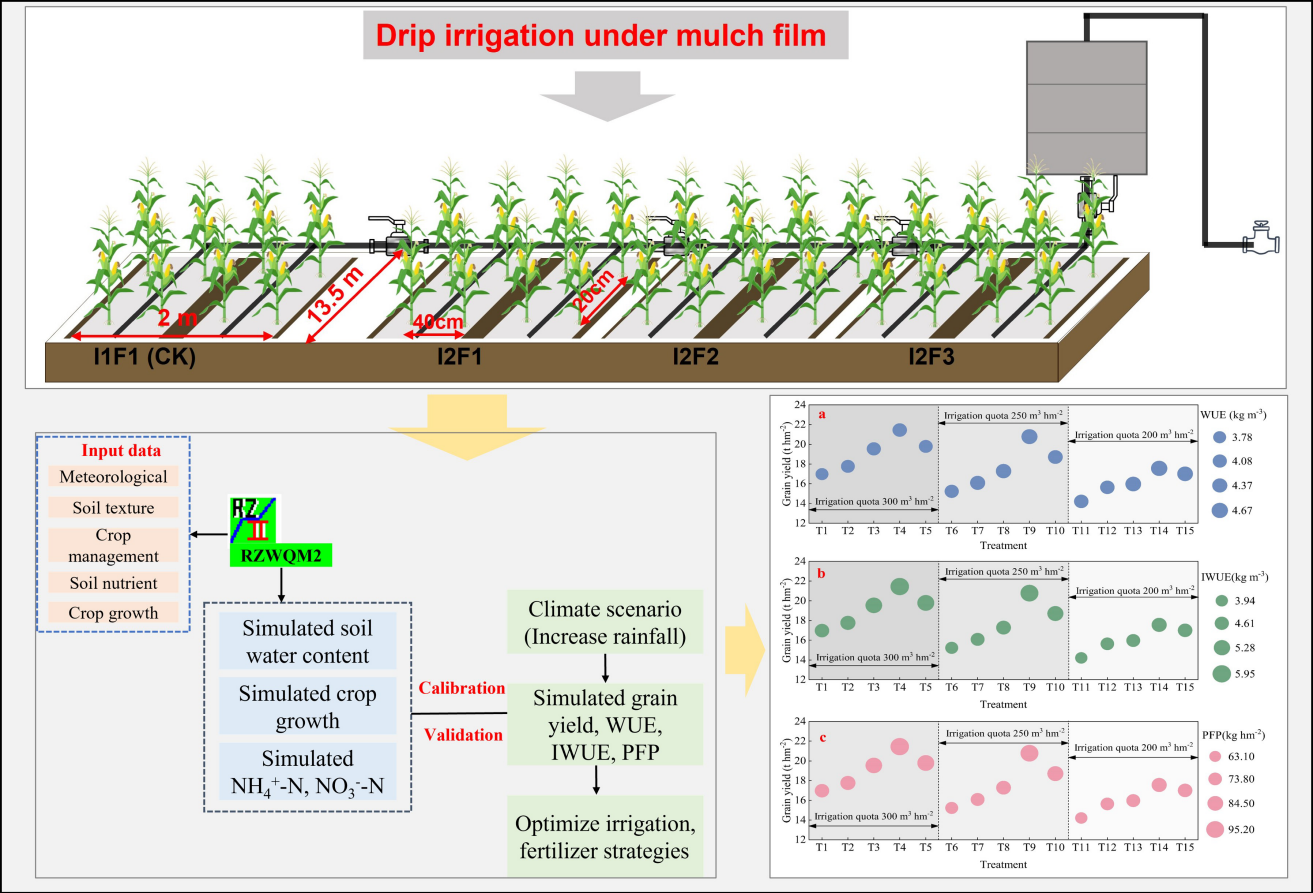
Schematic illustration of the study area.

**Figure 2:**
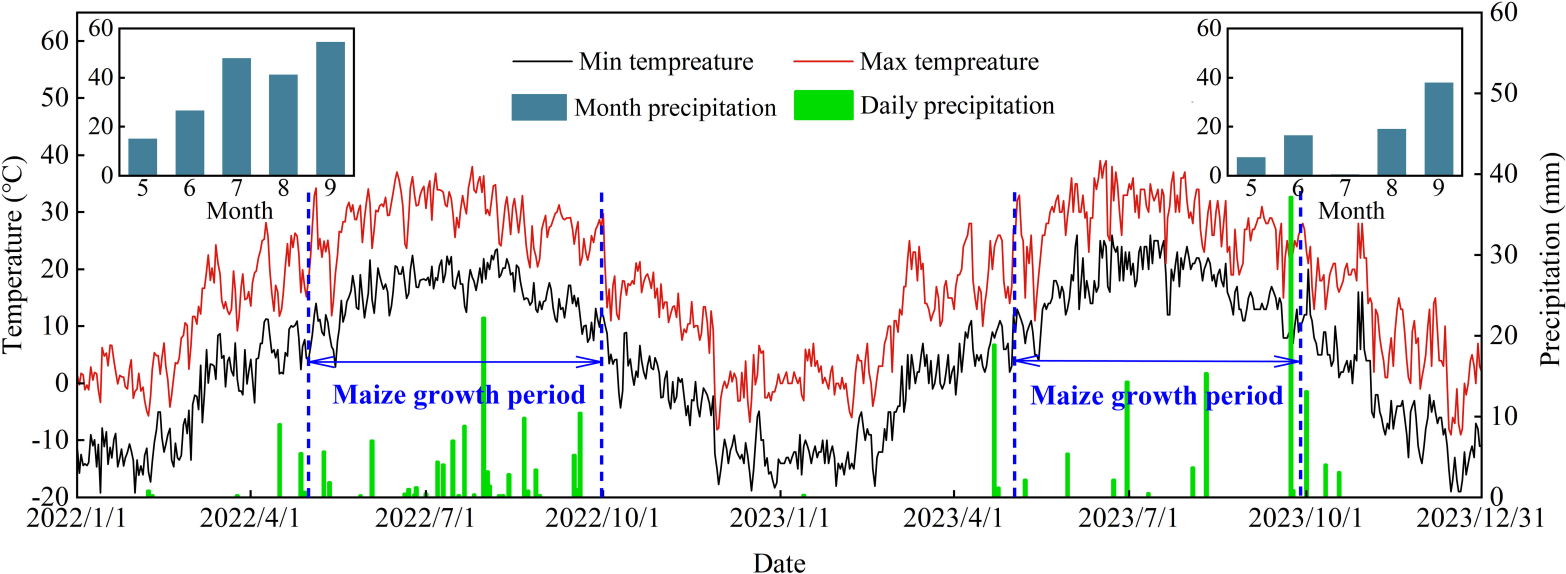
Precipitation, maximum temperature (T*max*), and minimum temperature (T*min*) during the spring maize growth season in 2022 and 2023.

The field experiment was conducted from 2022 to 2024 at the Minqin Experiment Station of the Gansu Research Institute of Water Conservancy (38°65′ N, 103°15′ E), China. The study site is located in the Hexi Corridor of Northeastern China (**Figure 1**), which has a temperate continental arid climate with an altitude of 1,580 m. The annual mean temperature, potential evaporation, and sunshine hours are approximately 7.8°C, 140 mm, 2,000 mm, and 3,000 h, respectively. In this study, the soil type was loamy, and the percentage of soil textures sandy, loamy, and clayey under the 0–60-cm soil layer was 38.1%, 41.3%, and 20.6%, respectively. Soil available phosphorus, potassium, potassium nitrate, ammonia nitrogen, and organic matter contents in the 0–20-cm soil layer were 29.56, 152.62, 40.23, 54.63, and 3.39 g kg^−1^, respectively. The precipitation during the spring maize growing seasons of 2022–2023 and 2023–2024 were 114.9 and 144.7 mm, respectively (**Figure 2**).

### 2.2 Field experiments

Six irrigation and fertilizer treatments were considered during the 2022 and 2023 field experiment periods. Each treatment had three replications, the plot had a size of 13.5 m × 2.0 m, and a 0.008-mm-thick white film was laid in one film with a four-row pattern (**Figure 1**). The irrigation fertilizer treatments included two different irrigation volumes (in this study, irrigation was performed when soil moisture values decreased to the lower limit of field capacity) [i.e., 75%–95% field capacity (I1) and 55%–75% field capacity (I2)] and three different fertilizer application rates [i.e., (N+P_2_O_5_ ≥ 64.0%) 234.37 kg hm^−2^ nitrogen fertilizer (F1), (N+P_2_O_5_ ≥ 64.0%) 157.50 kg hm^−2^ nitrogen fertilizer (F2), and (N+P_2_O_5_ ≥ 64.0%) 157.50 kg hm^−2^ nitrogen fertilizer plus 63.00 kg hm^−2^ organic fertilizer (F3)]. In this study, the organic fertilizer dosage was maintained at 63 kg hm^−2^, with conventional nitrogen fertilization remaining the predominant nutrient supply strategy according to standard agricultural protocols. The main reason for choosing three different types of fertilizers in this experiment was to investigate the changes in the growth of crops based on our normal application of nitrogen fertilizers and the addition of organic fertilizers to nitrogen reduction. Therefore, the four different water fertilizer treatments were I1F1, I2F1, I2F2, and I2F3 (**Table 1**). Drip irrigation was used during the spring maize growing seasons, and the irrigation amount was controlled using water metering devices. “Tong Kang DK818”, a local spring maize cultivar grown in the Northwest Arid Regions, was the spring maize variety used in this experiment. Seeds were over-sown manually with 40-cm row spacing on 1 May and 25 April in 2022 and 2023, respectively. At the seedling period, the planting density was approximately 80,000 plants ha^−1^. The spring maize was harvested on 30 September 2022 and 28 September 2023. Other field management practices were consistent with local management practices.

**Table 1:** Irrigation levels (I) and fertilizer rates (F) for spring maize during the growing season in 2022 and 2023.

Treatment	Irrigation treatment	Irrigation level	Fertilizer treatment	Fertilizer rate (kg hm^−2^)
I1F1 (CK)	I1 (adequate irrigation)	75%–95% $\theta_{f}$	F1 (nitrogen fertilizer)	234.37
I2F1	I2 (irrigation stress)	55%–75% $\theta_{f}$	F1 (nitrogen fertilizer)	234.37
I2F2	I2 (irrigation stress)	55%–75% $\theta_{f}$	F2 (nitrogen fertilizer)	157.50
I2F3	I2 (irrigation stress)	55%–75% $\theta_{f}$	F3 (nitrogen fertilizer +organic fertilizer)	157.50 + 63

$\theta_{f}$ is the soil field capacity.

**Figure 3:**
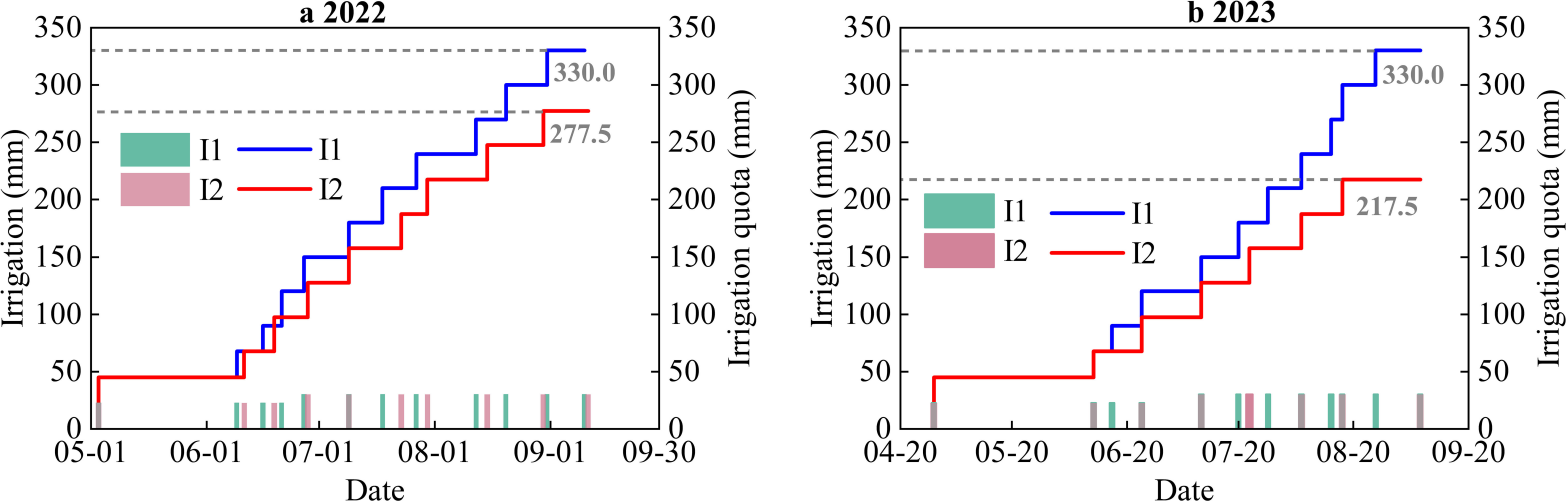
Cumulative irrigation and irrigation quota during the complete growth period of spring maize in 2022 **(a)** and 2023 **(b)**.

After spring maize was planted, each treatment by a (N+P_2_O_5_ ≥ 64.0%) 234.37 kg hm^−2^ fertilizer was applied at the ratio of 3:4:3 at the late seedling, nodulation, and filling stages, respectively. Before spring maize was planted, drip irrigation mains and branches were installed, with individual meters and valves for each treatment to control the amount of irrigation applied. The field capacity (*θ_f_*) of the experimental site was determined to be 20.75% by using the “frame flooding irrigation method” and volumetric water content determination method, with the upper limit of full irrigation at 95% *θ_f_* and the lower limit at 75% *θ_f_*, and the upper limit of partially flooded water at 75% *θ_f_* and the lower limit at 55% *θ_f_*. The irrigation was carried out when the relative water content of the soil decreased to the lower limit of water control. When the relative water content of the soil dropped to the lower limit of water control, irrigation was carried out. The cumulative amounts of adequate and stress irrigation were 330.0, 277.5, 330.0, and 217.5 mm during the complete growth period of spring maize in 2022 and 2023 (**Figure 3**). In this study, soil depth for irrigation management was 40 mm at the seedling stage and 60 mm at the nodulation to maturity stage in 2022 and 2023.

### 2.3 Data collection

#### 2.3.1 Meteorological data

The meteorological data during the spring maize growth period were measured from the China Meteorological Data Service Center (http://data.cma.cn, accessed on 29 January 2024) in this experiment site and included daily maximum and minimum temperature (°C), daily average temperature (°C), precipitation (mm), relative humidity (%), wind speed (km day^−1^), and sunshine hours (h). The daily radiation was determined by an integrated module based on the Penman–Monteith formula (Harmsen and Justiniano, 2016).

#### 2.3.2 Soil water content

Soil water content (SWC) was determined at a depth of 60 cm below the surface by the soil drying method. Soil samples were taken near the center of each plot every 5–7 days (after precipitation or irrigation) using a 3.5-cm-diameter soil auger, and samples were taken at 20-cm intervals below the surface. Wet soil samples were weighed and placed in an oven at 105°C for 8 h before being weighed.

#### 2.3.3 Yield

In each plot, the ears of spring maize were threshed after harvest, air-dried, and weighed, and the actual yield was determined by repeating the process three times. Five representative ears were randomly selected and brought back to the laboratory for seed testing to examine the number of rows, the number of grains, and the quality of 100 grains in each ear.

#### 2.3.4 Soil profile nitrate-N and ammonium-N contents

Soil nitrate nitrogen and ammonium nitrogen contents in the 0–20-cm, 20–40-, and 40–60-cm soil layers were determined at the seedling stage, elongation stage, silking stage, grain filling stage, and maturity stage of spring maize in 2022 and 2023. The nitrate-N (NO_3_^−^-N) and ammonium-N (NH_4_^+^-N) contents were determined by leaching with KCl solution (2 mol L^−1^ KCl solution, 5 g of dry soil, soil to solution ratio = 1:10) and using a UV-Vis spectrophotometer (Pulsar T6 New Century).

### 2.4 RZWQM2 description

The RZWQM2 is a one-dimensional model of agricultural systems and resource management introduced by the American Research Institute of Agricultural Systems (ARS) in 1992 (Ma et al., 2012a, b). The model consists of six sub-modules, including the nutrient cycling module, physical transport module, chemical reaction module, pesticide module, crop growth module, and management module, and each module interacts with each other (Ahuja et al., 2000).

The latest version of the RZWQM2 has evolved steadily since its debut in 1992 (Ahuja et al., 2000; Ma et al., 2007; Qi et al., 2012). The infiltration process of soil water was described in the model by the Green–Ampt infiltration equation, while Poiseuille’s and Richard’s equations were used to simulate macroporous flow and the redistribution of soil water, respectively (Ma et al., 2006; Jiang et al., 2020). The RZWQM2 offers a wide range of options for simulating crop growth, including the universal crop growth model, the CROPGRO model, the CERES model, the rapid planting crop model, the quick turf model, and the rapid tree model (Sadhukhan et al., 2019b, 2019a). In this study, the CERES-maize model in the crop module was selected to simulate maize growth and development, and the model crop parameters are shown in **Table 2**. Meanwhile, the data to be input into the model include meteorological data, initial soil conditions, soil characterization parameters, and field management data; the required soil physicochemical parameters are shown in **Table 3**. The nutrient process submodel (OMNI) was used to simulate changes in the nitrate (NO_3_^−^-N) and ammonium (NH_4_^+^-N) nitrogen contents of the soil profile using the carbon-to-nitrogen ratio (Shaffer et al., 1992). The initial values of different C/N pools and the C/N conversion factor were determined to improve the accuracy of the model in simulating soil NO_3_^−^-N and NH_4_^+^-N contents (Fang et al., 2008).

**Table 2:** Initial and calibrated values of crop genetic coefficients used for spring maize by RZWQM2.

Maize parameters	Definition	Calibrated values
P1 (°C days)	Thermal time from seeding emergence to the end of the juvenile phase	154.0 (100–400)
P2 (days)	Delay in development for each hour that daylength is above 12.5 h	0.48 (0–1)
P5 (°C days)	Thermal time from silking to physiological maturity	891.0 (600–1,000)
G2 (#)	Maximum possible number of kernels per plant	700.0 (500–1,000)
G3 (mg kernel day)	Kernel filling rate during the linear grain filling stage and under optimum conditions	7.0 (5–12)
PHINT (°C days)	The interval in thermal time between successive leaf tip appearances	45.0 (30–75)

**Table 3:** Main physical parameters of different soil depths in the experimental site.

Soil depth (cm)	Soil texture (%)			Bulk density (g cm^−3^)	Saturated hydraulic conductivity (cm h^−1^)	Field capacity (cm^3^ cm^−3^)
	Sand	Silt	Clay			
0–20	39.0	41.7	19.3	1.531	4.89	0.1854
20–40	38.3	41.3	20.4	1.523	3.55	0.1895
40–60	37.0	40.7	22.3	1.537	2.07	0.1879

### 2.5 Model performance criteria and calculations

The evaluation indicators of the model simulation were utilized in the study region (Nash and Sutcliffe, 1970), including root mean squared error (*RMSE*) (Equation 1), normalized root mean squared error (*NRMSE*) (Equation 2), mean relative error (*MRE*) (Equation 3), and consistency index (*d*) (Equation 4):


(1) $$RMSE=\sqrt{\sum_{i=1}^{N}\frac{{\left(S_{i}-O_{i}\right)}^{2}}{N}}$$

(2) $$NRMSE=\frac{1}{o_{avg}}\sqrt{\sum_{i=1}^{N}\frac{{\left(S_{i}-O_{i}\right)}^{2}}{N}}\times 100\%$$

(3) $$MRE=\frac{1}{N}\sum_{i=1}^{N}\left|\frac{S_{i}-O_{i}}{O_{i}}\right|\times 100\%$$

(4) $$d=1-\left[\frac{\sum_{i=1}^{n}{\left(S_{i}-O_{i}\right)}^{2}}{\sum_{i=1}^{n}{\left(\left|S_{i}-O_{avg}\right|+\left|O_{i}-O_{avg}\right|\right)}^{2}}\right]$$

where *S* is the simulated value, *O* is the observed value, *N* is the number of data points, *S_avg_* is the average of the simulated value, *O_avg_* is the average of the observed value, and *i* is the specific number of simulated and measured values.

*RMSE* reflects the magnitude of the average difference between the measured and simulated results (Gupta Hoshin et al., 1999), and *MRE* reflects the average value of the relative error. The closer the values of the *RMSE* and *MRE* are to 0, the more accurate is the model (Hong et al., 2021). *NRMSE* reflects the performance of the model (excellent is *NRMSE* ≤ 10%, good is 10% < *NRMSE* ≤ 20%, fair is 20% < *NRMSE* ≤ 30%, and poor is *NRMSE* > 30%) (Jamieson et al., 1991). A perfect match between the measurement results and simulation results would yield an *NRMSE* of 0 (Zhang et al., 2023). *d* determines the degree of correlation between the measured and simulated results. The closer the value of *d* is to 1, the more accurate is the model (Moriasi et al., 2015).

### 2.6 Irrigation quota and fertilizer scenario design

In this study, to determine the appropriate irrigation and fertilizer scheduling for spring maize, three irrigation quotas (300, 250, and 200 m^3^ hm^−2^) and five fertilizer applications (225 kg hm^−2^ organic fertilizer, 45 kg hm^−2^ nitrogen fertilizer and 180 kg hm^−2^ organic fertilizer, 90 kg hm^−2^ nitrogen fertilizer and 135 kg hm^−2^ organic fertilizer, 135 kg hm^−2^ nitrogen fertilizer and 90 kg hm^−2^ organic fertilizer, and 180 kg hm^−2^ nitrogen fertilizer and 45 kg hm^−2^ organic fertilizer) with 15 years (2009–2023) of 15-mm increase in rainfall, changes in water use efficiency (*WUE*) (Equation 5), irrigation water use efficiency (*IWUE*) (Equation 6), and partial factor productivity (*PFP*) (Equation 7) were investigated (**Table 4**). Meanwhile, the number of irrigations was set to 12, and the irrigation dates were set based on local precipitation variations. The calibrated and validated RZWQM2 model was used to simulate and calculate the yield, *WUE*, *IWUE*, and *PFP* for each scenario. Then, the appropriate irrigation quota and fertilizer amount were determined as those with a high yield, *WUE*, *IWUE*, and *PFP* by analyzing these indicators.

**Table 4:** Design of simulated scenarios with different irrigation quotas and fertilizer applications by spring maize.

Scenario treatment	Irrigation quota (m^3^ hm^−2^)	Fertilizer type	Fertilizer application (kg hm^−2^)
T1	300	Organic fertilizer 100%	225
T2		Nitrogen fertilizer 20% and organic fertilizer 80%	45 + 180
T3		Nitrogen fertilizer 40% and organic fertilizer 60%	90 + 135
T4		Nitrogen fertilizer 60% and organic fertilizer 40%	135 + 90
T5		Nitrogen fertilizer 80% and organic fertilizer 20%	180 + 45
T6	250	Organic fertilizer 100%	225
T7		Nitrogen fertilizer 20% and organic fertilizer 80%	45 + 180
T8		Nitrogen fertilizer 40% and organic fertilizer 60%	90 + 135
T9		Nitrogen fertilizer 60% and organic fertilizer 40%	135 + 90
T10		Nitrogen fertilizer 80% and organic fertilizer 20%	180 + 45
T11	200	Organic fertilizer 100%	225
T12		Nitrogen fertilizer 20% and organic fertilizer 80%	45 + 180
T13		Nitrogen fertilizer 40% and organic fertilizer 60%	90 + 135
T14		Nitrogen fertilizer 60% and organic fertilizer 40%	135 + 90
T15		Nitrogen fertilizer 80% and organic fertilizer 20%	180 + 45


(5) $$WUE=\frac{Y_{maize}}{ET}$$

(6) $$IWUE=\frac{Y_{maize}}{I_{irrigation}}$$

(7) $$PFP=\frac{Y_{maize}}{F_{fertilizer}}$$

where *Y_maize_* is the simulated spring maize grain yield (t hm^−2^), *ET* is the simulated evapotranspiration in spring maize growth period (mm), *I_irrigation_* is the irrigation quota (m^3^ hm^−2^), and *F_fertilizer_* is the fertilizer application (kg hm^−2^).

### 2.7 Statistical analysis

Excel 2016 and Minitab 15 were used for data processing, and Origin 2024 was utilized to create figures in this study. Soil water content and crop biomass data are expressed as means of three replications. Data were analyzed for significant differences using the IBM SPSS Statistics 27 statistical software. Multiple comparisons were performed using the least significant difference (LSD) test at *p* < 0.05.

## 3 Results

### 3.1 Model calibration and validation of soil water content and crop growth

**Figure 4:**
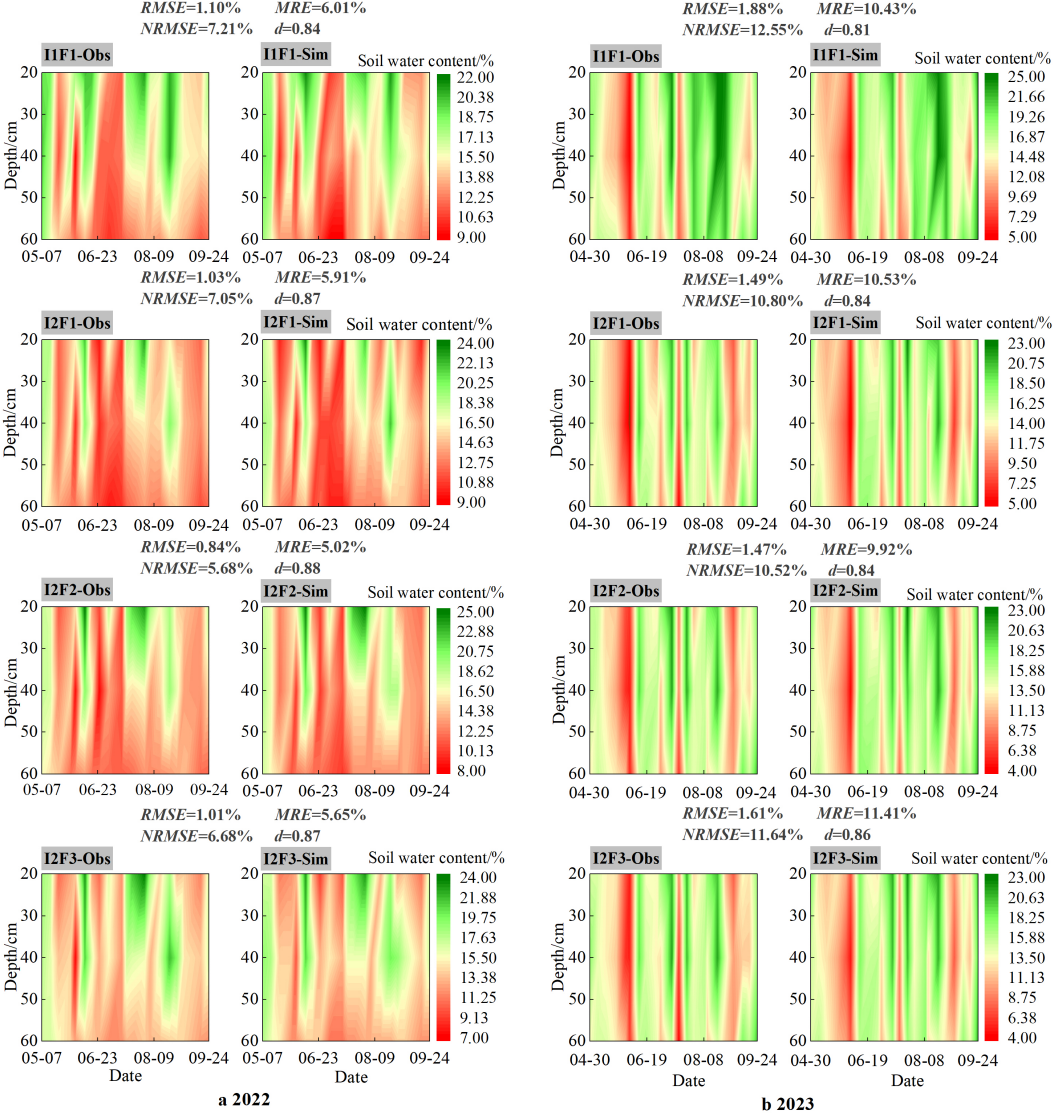
Contour maps of observed and simulated soil water content (SWC) at 0–60-cm depth under four treatments during the growth period of spring maize in 2022 **(a)** and 2023 **(b)**. Note: I1F1, I2F1, I2F2, and I2F3 stand for adequate irrigation and traditional fertilizer, irrigation stress and traditional fertilizer, irrigation stress and traditional fertilizer reduction, and irrigation stress and traditional fertilizer reduction plus organic fertilizer, respectively. *RMSE* is root mean squared error, *NRMSE* is normalized root mean squared error, *MRE* is mean relative error, and *d* is consistency index.

**Figure 4** shows the variation in the observed and simulated SWC values in the 0–60-cm soil layer for different treatments during 2022 and 2023. In this study, measured SWC data from 2022 were used to calibrate the model parameters, and the model from 2023 was validated. In the whole growth seasons, the observed and simulated values of SWC under the four treatments were relatively similar and corresponded to the trend of the SWC dynamics of spring maize in 2022 and 2023.

The *RMSE* values of SWC in the 0–60-cm soil layer under the four treatments were in the range of 0.84%–1.10% and 1.47%–1.88% in 2022 and 2023, respectively. The *NRMSE* and *MRE* values for the 0–60-cm layer were below 15.00% in 2022 and 2023. The calibrated *d* values of the simulated SWC were 0.84, 0.87, 0.88, and 0.87 under the four treatments for the 0–60-cm layers in 2022. Meanwhile, the validated *d* values of simulated SWC were greater than 0.80 under the four treatments in 2023 of the spring maize growth period. Overall, the RZWQM2 showed good applicability in simulating spring maize SWC in this region.

### 3.2 Model calibration and validation of soil water content and crop growth of biomass

**Figure 5:**
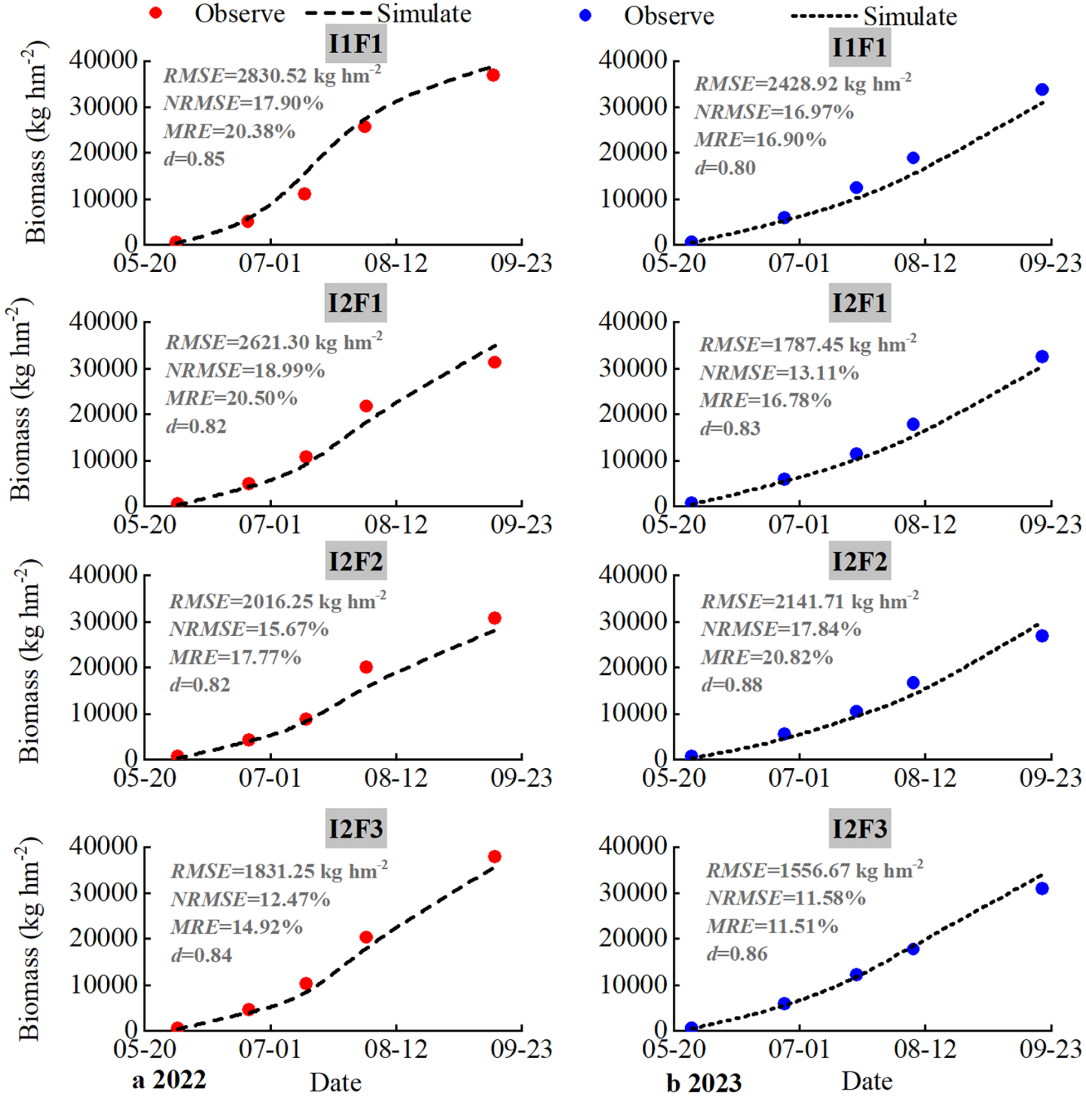
Comparison between observed and simulated biomass under different treatments during the growth period of spring maize in 2022 **(a)** and 2023 **(b)**. I1F1, I2F1, I2F2, and I2F3 stand for adequate irrigation and nitrogen fertilizer, irrigation stress and nitrogen fertilizer, irrigation stress and nitrogen fertilizer reduction, and irrigation stress and nitrogen fertilizer reduction plus organic fertilizer, respectively. *RMSE* is root mean squared error, *NRMSE* is normalized root mean squared error, *MRE* is mean relative error, and *d* is consistency index.

Across both calibration (2022) and validation (2023) phases, spring maize biomass values obtained through field measurements and model simulations exhibited congruent incremental patterns during the entire growth cycle under four experimental treatments, demonstrating strong consistency between simulated and observed crop parameters (**Figure 5**). Maize biomass showed a trend of cumulative increase as fertility advances, with biomass values peaking at the end of the growth period.

The simulated results of spring maize biomass under the four treatments’ *RMSE*, *NRMSE*, *MRE*, and *d* values were in the range of 1,831.25–2,830.52 kg hm^−2^, 12.47%–18.99%, 14.92%–20.50%, and 0.82–0.85, respectively, in the calibration period (2022). The simulated results of spring maize biomass under the four treatments’ *NRMSE* and *MRE* values were maize biomass simulations that resulted in *NRMSE* and *MRE* values less than 20% and *d* values greater than 0.80 in the validation period (2022). For spring maize biomass, the simulated biomass dynamics for four treatments by the RZWQM2 were consistent with their observed values during the whole growth seasons. Overall, for the spring maize biomass data, the simulated results acceptably matched the observed values, suggesting that the RZWQM2 could be used to accurately describe the crop growth processes under the irrigation and fertilizer conditions.

### 3.3 Model calibration and validation of spring maize grain yield

**Figure 6:**
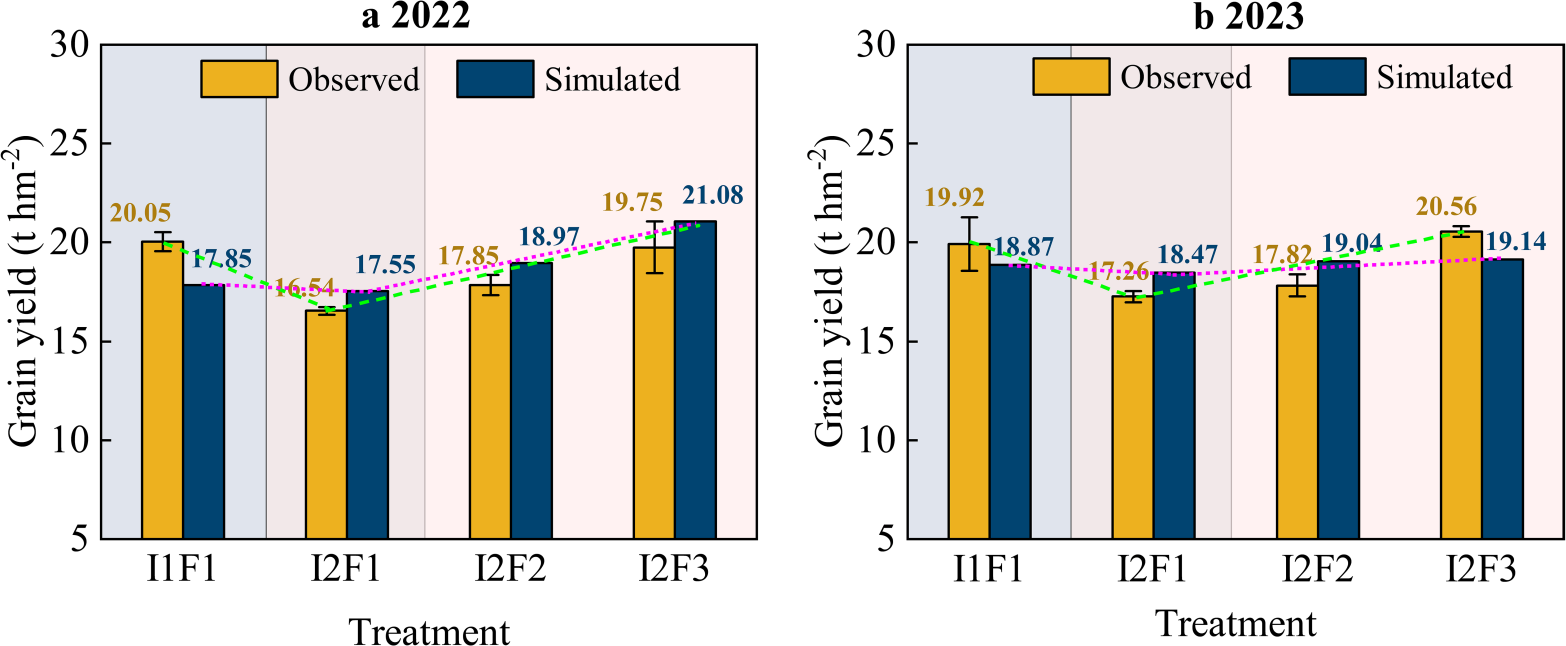
Comparison between observed and simulated grain yield under different treatments of spring maize in 2022 **(a)** and 2023 **(b)**. Error bars indicate standard deviation (*p* < 0.05). Note: I1F1, I2F1, I2F2, and I2F3 stand for adequate irrigation and nitrogen fertilizer, irrigation stress and nitrogen fertilizer, irrigation stress and nitrogen fertilizer reduction, and irrigation stress and nitrogen fertilizer reduction plus organic fertilizer, respectively.

**Figure 6** illustrates the spring maize grain yield trends and the results of observed and simulated values under the four treatments in 2022 and 2023. Under the I2F3 treatment, observed spring maize grain yields were higher than those under the I1F1 treatment, and the simulated values showed consistent results in both 2022 and 2023. However, there was no significant difference in yield between the I1F1 and I2F3 treatments (*p* > 0.05). Both observed and simulated spring maize drain yield values under the I2F1, I2F2, and I2F3 treatments generally showed an upward trend in both 2022 and 2023. Compared with the I1F1 treatment, observed spring maize grain yield under the I2F3 treatment significantly increased by 16.25% and 16.05% in 2022 and 2023 (*p* < 0.05), respectively. The *RMSE* and *MRE* values for four treatments of spring maize grain yield simulated were better in the RZWQM2 in 2022 and 2023 (**Figure 6**). In 2022 and 2023, the simulated results of spring maize grain yield under the four treatments’ *RMSE* and *MRE* values were in the range of 1.12–2.47 t hm^−2^ and 6.27%–12.31%, and 1.05–1.42 t hm^−2^ and 5.28%–6.98%, respectively. These results indicate that the RZWQM2 could better simulate the variation in spring maize grain yield.

### 3.4 Model calibration and validation of soil NH_4_^+^-N and NO_3_^−^-N dynamics

**Figure 7:**
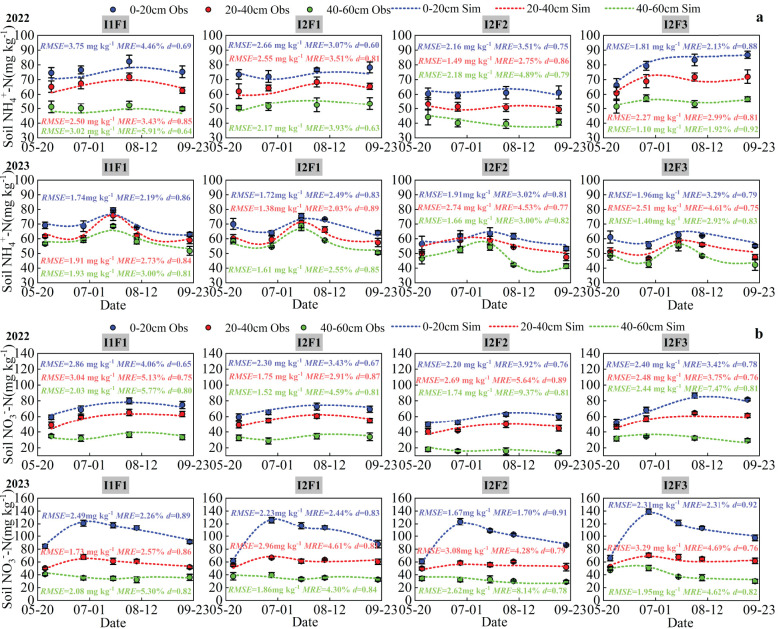
Comparison between observed and simulated soil NH_4_^+^-N**(a)** and NO_3_^−^-N**(b)** contents under different treatments of spring maize in 2022 and 2023. Error bars indicate standard deviation (*p* < 0.05). I1F1, I2F1, I2F2, and I2F3 stand for adequate irrigation and nitrogen fertilizer, irrigation stress and nitrogen fertilizer, irrigation stress and nitrogen fertilizer reduction, and irrigation stress and nitrogen fertilizer reduction plus organic fertilizer, respectively. *RMSE* is root mean squared error, *MRE* is mean relative error, and *d* is consistency index.

Simulated soil NH_4_^+^-N and NO_3_^−^-N contents over time generally followed the observed values at the four treatments’ soil depths (0–20, 20–40, and 40–60 cm) for the calibration (2022) and validation data (2023) (**Figure 7**). Dynamics in simulated soil NH_4_^+^-N and NO_3_^−^-N contents in different soil depths during the growth period of spring maize under the four treatments were consistent with the simulated trend in 2022 and 2023. The soil NH_4_^+^-N and NO_3_^−^-N contents at different soil depths under all four treatments were 0–20 cm > 20–40 cm > 40–60 cm of spring maize growth period in 2022 and 2023. The observed values of soil NH_4_^+^-N and NO_3_^−^-N contents of I1F1, I2F1, I2F2, and I2F3 were not significantly different between treatments (*p* > 0.05) in 2023. Compared with the I2F2 treatment, the I1F1, I2F1, and I2F3 treatments significantly increased average soil NH_4_^+^-N content (0–20, 20–40, and 40–60 cm) by 21.59%, 20.51%, and 24.23% in 2022 (*p* < 0.05), respectively. Compared with the I2F2 treatment, the I1F1, I2F1, and I2F3 treatments significantly increased average soil NO_3_^−^-N content (0–20, 20–40, and 40–60 cm) by 29.61%, 23.53%, and 27.26% in 2022 (*p* < 0.05), respectively.

For simulated soil NH_4_^+^-N and NO_3_^−^-N contents in each soil layer, the *RMSE* values were between 1.10 and 3.75 mg kg^−1^, the *MRE* values were between 1.92% and 4.89%, the *NRMSE* values were between 2.01% and 5.93%, and the *d* values were between 0.60 and 0.92 under the four treatments in 2022. The mean *RMSE*, *MRE*, *NRMSE*, and *d* values of the NH_4_^+^-N and NO_3_^−^-N content simulations across the four treatments were 2.00 mg kg^−1^, 2.46%, 2.50%, and 0.86 in the 0–20-cm soil layer, respectively, in spring maize growth period. The values in the 0–20-cm soil layer were 2.45 mg kg^−1^, 3.76%, 4.21%, and 0.83, respectively. The values in the 20–40-cm soil layer were 1.89 mg kg^−1^, 4.23%, 4.59%, and 0.82, respectively. Overall, these results indicate that the simulation of NH_4_^+^-N and NO_3_^−^-N contents by the RZWQM2 model has better applicability during the growth period of spring maize in this study region.

### 3.5 Crop yield, water use efficiency, irrigation water use efficiency, and partial factor productivity under climate change scenarios (2009–2023)

**Figure 8:**
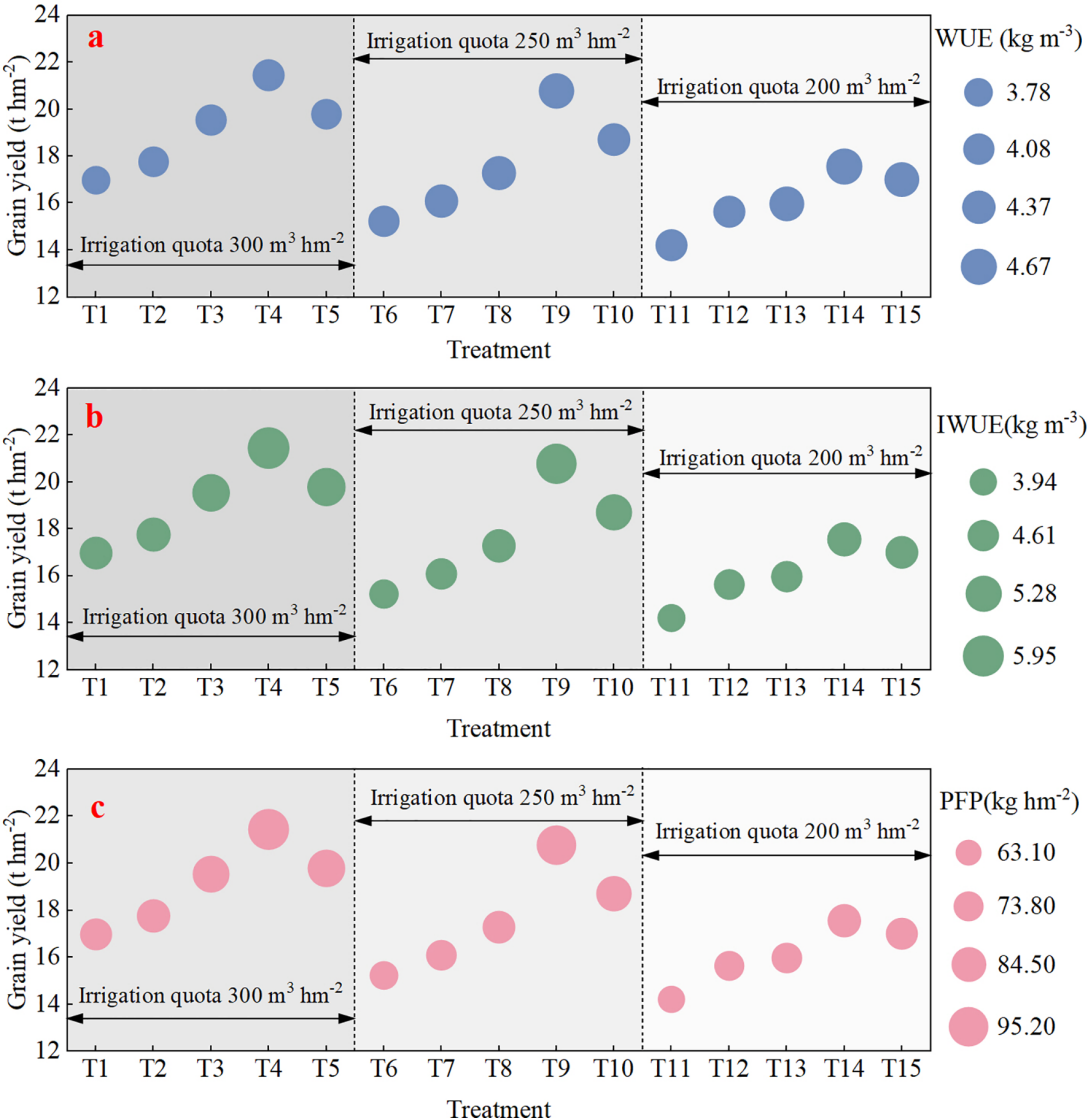
Spring maize yield, water use efficiency (*WUE* **a)**, irrigation water use efficiency (*IWUE* **b)**, and partial factor productivity (*PFP* **c)** under different combined water and fertilizer treatments in climate change scenarios. Treatments T1–T15 represent three irrigation quotas (300, 250, and 200 m^3^ hm^−2^) and five fertilizer applications (225 kg hm^−2^ organic fertilizer, 45 kg hm^−2^ nitrogen fertilizer and 180 kg hm^−2^ organic fertilizer, 90 kg hm^−2^ nitrogen fertilizer and 135 kg hm^−2^ organic fertilizer, 135 kg hm^−2^ nitrogen fertilizer and 90 kg hm^−2^ organic fertilizer, and 180 kg hm^−2^ nitrogen fertilizer and 45 kg hm^−2^ organic fertilizer).

Spring maize yield, *WUE*, *IWUE*, and *PFP* for different irrigation and fertilizer scenarios are shown in **Figure 8**. The yield, *WUE*, *IWUE*, and *PFP* showed an increasing trend and then a decreasing trend at the three different irrigation quotas. Spring maize yields showed maximum performance under the 60% nitrogen fertilizer and 40% organic fertilizer scenarios in three irrigation quotas (300, 250, and 200 m^3^ hm^−2^) of 21.45, 20.78, and 17.57 t hm^−2^, respectively. *WUE* values were greater under the T9 and T14 treatments at 4.56 and 4.67 kg m^−3^, respectively. The different irrigation and fertilizer treatments with simulated larger values of *IWUE* were T9, T14, and T15 with 5.96, 5.77, and 4.88 kg m^−3^, respectively. The *PFP* value was greater under the T4 and T9 treatments with 95.33 and 92.36 kg kg^−1^, respectively. In summary, the optimal irrigation and fertilizer coupling strategy for spring maize under climate change scenarios was an irrigation quota of 300 m^3^ hm^−2^ and 60% nitrogen fertilizer and 40% organic fertilizer (T9).

## 4 Discussion

### 4.1 Model evaluation of soil water content, biomass, nitrate-N, ammonium-N, and grain yield in spring maize

In this study, the soil water content values of different soil layers (0–60 cm) were simulated to be consistent with observations, with *MRE* and *NRMSE* values less than 15% for all treatments and *d* values greater than 0.8 for both growth seasons (**Figure 4**). However, the model simulated lower values for full irrigation results than for the irrigation stress treatment. This is probably because the model has a warm-up process in the early stages of the simulation (Fang et al., 2013). Meanwhile, the results of the simulated values of soil water content were lower in the validation period (2023) than in the calibration period (2022), which may be attributed to the low simulation results due to the meteorological conditions in 2023 when the precipitation was lower than in 2022 (Ma et al., 2012).

In conclusion, the RZWQM2 model simulated spring maize grain yield (*MRE* values ranged from 5.28% to 12.31%) and aboveground biomass (*MRE* values ranged from 11.51% to 20.82%, with *d*-values greater than 0.85) better (**Figure 6**). Zhang et al. (2021) found that the calibrated RZWQM2 model was able to accurately simulate the yield and above-ground biomass of maize, and the simulation results with *R* ^2^ ranged from 0.74 to 0.85, *NRMSE* < 15%. Sima et al. (2019) evaluated that the RZWQM2 model could provide a better simulation of the yield and biomass of irrigated maize in Eastern Colorado. This was similar to the results in this study where all *d* values of maize biomass were greater than 0.80 and maize yield *MRE* values were less than 10.00% as simulated by the RZWQM2 model. Therefore, it was further confirmed that the model has a better applicability for the simulation of maize yield and biomass.

The RZWQM2 model performed well for the fitting of NH_4_^+^-N and NO_3_^−^-N under different soil layers (0–20, 20–40, and 40–60 cm), with *RMSE* values less than 5.00 mg kg^−1^, *MRE* and *NRMSE* values less than 10%, and *d* values greater than 0.60 (**Figure 7**). Ding et al. (2020) used the RZWQM2 model for soil NH_4_^+^-N and NO_3_^−^-N contents for different tillage systems with consistency index *d* values ranging from 0.71 to 0.93. In this study, the fluctuation of nitrogen dynamics during the growth stage of maize was smaller in 2023 than in 2022 (**Figure 7**). This was probably because the precipitation was greater in 2022 (185.09 mm) than in 2023 (81.31 mm) during the spring maize growth period. The dynamics of NH_4_^+^-N and NO_3_^−^-N in different soil layers were strongly influenced by irrigation and rainfall (Sato et al., 2009; Allaire-Leung et al., 2001). Ding et al. (2024) also confirmed in their study that fluctuations in nitrogen content changes were greater in wet years than in dry years.

In summary, the calibrated RZWQM2 model performed well under different irrigation and fertilizer conditions, providing a promising tool for determining the optimal irrigation and fertilizer coupling for spring maize.

### 4.2 Optimization of spring maize irrigation quota and fertilizer application rate using the RZWQM2 model

In recent years, the continued warming of the climate has led to the expansion of the westerly wind belt, a progressively wetter climate, and a gradual trend toward increased precipitation (Li et al., 2024; Zheng et al., 2021). Sridevi et al. (2024) based on the DSSAT model enabled a 10% increase in maize yield to future temperature increases in climatic conditions and obtained an optimal fertilization program combining inorganic and organic fertilizers. In this study, similar results were obtained by increasing the temperature by 0.5°C to obtain an 18.08% higher maize yield. Ding et al. (2024) evaluated the optimal nitrogen fertilizer measurement for maize under different precipitation conditions through the RZWQM2 model and obtained higher maize growth, yield, and nitrogen fertilizer use efficiency, with future precipitation increased to 300 mm. In this study, the agronomic optimal irrigation–fertilization regime was determined through a multi-criterion assessment integrating grain yield maximization with improvements in *WUE*, irrigation water use efficiency (*IWUE*), and partial factor productivity (*PFP*) (**Figure 8a–c**). However, the partial factor productivity value was higher at 95.33 kg kg^−1^ when the irrigation gradient was 300 m^3^ hm^−2^ fertilized with 60% nitrogen and 40% organic fertilizers, but reduced the *WUE* (4.18 kg m^−3^) and *IWUE* (71.50 kg m^−3^) (**Figure 8a**). In addition, when the irrigation gradient was 200 m^3^ hm^−2^ and the fertilizer was 60% nitrogen and 40% organic fertilizers, *WUE* (4.67 kg m^−3^) and *IWUE* (4.88 kg m^−3^) improved but *PFE* (78.09 kg kg^−1^) severely reduced (**Figures 8b,c**), and the probable reason was excessive water stress while reducing nitrogen leaching, inhibiting plant uptake (Laskari et al., 2022; Liao et al., 2022).

Meanwhile, previous studies have shown that the optimal inorganic-to-organic fertilizer ratio for maximizing maize yield is 3:2 (Zhou et al., 2021), which is consistent with the results of this study. The main reason is that a moderate reduction of nitrogen fertilizer can reduce nitrogen leaching, while a moderate increase in organic fertilizer can effectively enhance soil fertility (Han, 2022) and promote crop root growth to increase the root–shoot ratio (Wei et al., 2023), thereby enhancing crop yield. In conclusion, the optimum fertilizer rates for spring maize in this study area were 135 kg hm^−2^ nitrogen fertilizer and 90 kg hm^−2^ organic fertilizer. The results of combining nitrogen fertilizers were similar to those of Han (2022). In Han’s study, the 40% organic fertilizer replacement treatment had higher yields than the 50% organic fertilizer replacement and 60% organic fertilizer replacement treatments.

This study mainly focused on the semi-arid region with the objective of obtaining high-yielding and efficient spring maize, which resulted in certain shortcomings such as the neglect of the soil texture, changes in soil physicochemical properties, and the impact of economic efficiency on irrigation and fertilizer. Therefore, to determine the optimal irrigation quota and fertilizer application rate for maize, further studies should comprehensively consider the impacts of the soil environment, economic benefits, and ecological benefits.

## 5 Conclusion

In this study, the RZWQM2 model was calibrated and validated using observed data of soil water content, soil NH_4_^+^-N and NO_3_^−^-N contents, biomass, and grain yield from 2022 and 2023. Then, it was applied for scenario analysis considering climate change during 2009–2023, irrigation, and fertilizer rates. Results determined four soil parameters and six crop parameters for “Tong Kang DK818” spring maize in the RZWQM2 model. The model’s accuracy in simulating soil water content, maize biomass, N content, and grain yield was validated through calibration (2022) and validation (2023). The simulation accuracy of soil water content, soil NH_4_^+^-N and NO_3_^−^-N contents, spring maize biomass, and grain yield were satisfactory, as indicated by the *RMSE*, *NRMSE*, *MRE*, and *d* values for both 2022 and 2023. The calibrated RZWQM2 model was then used to determine optimal irrigation and fertilizer application rates for spring maize using 15 years of historical weather data. Simulation results showed that an irrigation quota of 300 m^3^ hm^−2^ combined with 60% nitrogen fertilizer and 40% organic fertilizer (135 + 90 kg hm^−2^ of T9 treatment) could achieve high grain yield, *WUE*, *IWUE*, and *PFP*. These results contribute to the development of a scientific irrigation and fertilizer management system for achieving high yield and high resource use efficiency in the spring maize production of the semi-arid region.

## Data Availability

The original contributions presented in the study are included in the article/supplementary material. Further inquiries can be directed to the corresponding author.
